# Perceptions of mental health, suicide and working conditions in the construction industry—A qualitative study

**DOI:** 10.1371/journal.pone.0307433

**Published:** 2024-07-24

**Authors:** Kristina Aurelius, Mia Söderberg, Viktoria Wahlström, Margda Waern, Anthony D. LaMontagne, Maria Åberg

**Affiliations:** 1 Occupational and Environmental Medicine, School of Public Health and Community Medicine, Sahlgrenska Academy, University of Gothenburg, Gothenburg, Sweden; 2 Occupational and Environmental Medicine, Sahlgrenska University Hospital, Region Västra Götaland, Gothenburg, Sweden; 3 Department of Public Health and Clinical Medicine, Umeå University, Umeå, Sweden; 4 Region Västra Götaland, Sahlgrenska University Hospital, Gothenburg, Sweden; 5 Department of Psychiatry and Neurochemistry, Institute of Neuroscience and Physiology, Sahlgrenska Academy, University of Gothenburg, Gothenburg, Sweden; 6 Melbourne School of Population and Global Health, The University of Melbourne, Melbourne, Australia; 7 Institute for Health Transformation & School of Health & Social Development, Deakin University, Geelong, Victoria, Australia; 8 Region Västra Götaland, Regionhälsan, Gothenburg, Sweden; University of Toronto, CANADA

## Abstract

**Objective:**

The aim of the study was to explore perceptions of mental ill health, suicidal behaviour and working conditions among male construction workers, in order to gain an in-depth understanding of these phenomenon and to identify relevant avenues for workplace interventions.

**Method:**

Data were collected in individual and group interviews, and 43 individuals from the Swedish construction industry, workers, union representative and managers, participated in the study. Inductive thematic analysis was used to analyse the data.

**Results:**

Five main themes were found: Difficult to talk about mental health, Demanding working environment affects mental health, Substance abuse among construction workers, Importance of management, and Need for routines and social support in the workplace. Many participants reported that there was a stigma related to mental health. Suicides that had occurred among colleagues were perceived to come out of the blue. The working environment in the construction industry was perceived to have a negative effect on mental health, and it was reported that the management played an important role in both the cause and prevention of mental health problems.

**Conclusions:**

The results from this Swedish study are in accordance with previous international research regarding a macho culture, stigma of mental health and a demanding working environment in the construction industry. The study adds to existing knowledge by highlighting that suicides were perceived to be very unexpected, that poor physical health affected mental health and that many participants did not know how to deal with mental health issues in the workplace.

## Introduction

Suicide is a global problem, and in Sweden approximately 1500 individuals die by suicide every year [1]. A number of risk factors for suicide have been identified including mental health disorders [2], somatic illness [3], unemployment and low socio-economic status [4]. Occupation and work-related factors also appear to influence the risk of suicide [1, 5, 6].

Adverse working conditions can potentially lead to suicide via depression or other psychiatric disorders and accumulating evidence shows that job stressors are associated with mental health problems [7, 8]. However, not all who die by suicide suffer from psychiatric disorders, and other work-related factors that have been associated with suicide include poor social support, bullying, exposure to trauma on the job, working in isolation and access to means [9].

There is growing evidence linking psychosocial job stressors to suicide [7, 9]. Milner et al [10] conducted a meta-analysis of suicidality and a wide range of psychosocial job stressors such as high demands, low control and lack of support, and found that exposure to job stressors was associated with an elevated risk of suicidal ideation and behaviours. The authors concluded that there is some evidence that psychosocial job stressors could be related to suicidal outcomes but highlighted that most studies were cross-sectional and that there was a need for further research to assess the robustness of the findings. A systematic literature review of work and suicide [5] found a link between occupational stressors and suicidal ideation. It was suggested that workplace ostracism, being excluded or ignored at work, may have a particularly strong effect on suicidal ideation, as this may lead to extreme feelings of thwarted belonginess [5, 11]. Workplace social support was associated with a lower risk of suicide [5]. Similarly, other studies of psychosocial factors at work have found an increased risk of suicidal behaviour for passive jobs (low demands and low control), low social support, high strain (high demands and low control) and low control [8, 9, 12]. In addition, recent research has suggested that gender dominance at workplace and occupation can be associated with suicide risk among men [1].

Death by suicide is more common among men than among women worldwide [13]. Men use more lethal suicide methods, are more likely to underreport mental ill-health and less often seek help from professionals than women [5, 14, 15]. Construction workers have an increased risk of work-related ill-health [16, 17], and male construction workers have been identified as a group with elevated risk of suicide [18–22]. A recent meta-analysis found an increased risk of construction worker suicide compared to the wider population, with a relative suicide risk of 1.25 [19]. In the US the suicide rates have been reported to be four times higher in the construction industry compared to the national average, and in the UK 3.7 times higher [22]. In Sweden it has been found that specific occupations within the construction sector, laborers and rock workers, have a higher risk for suicidal behaviour compared to other occupational groups within the construction sector, with hazard ratios of 1.4 and 1.5 respectively [6]. Poor help-seeking, stigma relating to mental health issues and macho culture have been identified as reasons to why males in the construction industry have a higher risk of suicide, although the reasons are multifaceted [20, 22]. In a recent literature review of suicide in the construction industry [17] the most common suicide risk factor was job insecurity followed by alcohol and substance abuse, lack of help-seeking, physical injury and chronic pain. Other possible reasons for higher risks for suicide among construction workers is lower educational attainment. Employees in construction have a lower educational attainment compared to most other industries [23]. A large study with more than 815 000 participants found that educational attainment may causally influence suicide attempt risk [24]. In addition, social isolation, financial insecurity and homelessness have been identified as suicide risk factors for construction workers [17]. A mixed-methods study [21] found that many construction workers do not share or seek help when in distress. They also found that there was an experience of distrust towards services provided by the workplace and that alcohol or drugs were used for self-medication against distress. On the other hand, these elevated rates may be modifiable: A study investigating the suicide mortality of Australian male construction workers during the last two decades found a rapid decline among construction workers compared to other workers [18]. The (surveillance) analysis did not address why this was the case, but the authors speculated that the improved economic activity in the construction industry, lower unemployment rates, sector-specific suicide prevention initiatives, and other factors may have contributed to the decline in suicide rates.

The workplace can be an arena for suicide prevention by focusing on workplace factors that influence the risk of suicide, including promoting helping behaviours, reducing stigma, and improving working conditions [5, 9, 22]. To inform future workplace interventions it is important to increase the knowledge of mental health and suicidal behaviour in the construction industry. Qualitative studies can provide in-depth investigations of complex phenomena such as mental health and suicidal behaviour in a work context. To our knowledge there are no previous Swedish qualitative studies investigating these issues among male construction workers in Sweden. The aim of the present study was to explore perceptions of mental ill health, suicidal behaviour and working conditions among male construction workers, in order to increase the understanding of these phenomenon and to identify relevant avenues to tailor workplace interventions to the Swedish context.

## Materials and methods

### Participants

A purposeful sampling strategy was used to ensure that the sample consisted of male participants with relevant experience relating to the study aims. In total, 43 males participated, with 11 individual interviews and 7 focus group interviews. The participants were individuals with work experience in the construction industry including workers, managers, and union representatives. The participants were recruited via different forums including unions, large and small construction companies, and advertisement in a construction industry union magazine, and they are further described in Table 1. The recruitment took place from 01/09/2022 until 01/05/2023. Participants received no reimbursement for their participation.

**Table 1:** Information regarding participants.

	N	Individual interviews	Focus groups	Mean age	Age-range
Workers	22	6 (3 digital)	3	36	20–53
Managers	10	1	2	40	26–54
Union representatives	11	4 (3 digital)	2	49	35–62
All	43	11	7	40	23–62

### Procedure and data collection

Focus group interviews were used as the primary method to collect the data. This method was used as focus group interviews is a flexible method that takes advantage of the participants communication with each other. The participants experiences and attitudes are explored, and the group interaction is part of the method [25]. Considering the sensitive nature of the subject area, participants could choose to participate in an individual interview. Digital interviews were an option, and 6 of the 11 individual interviews were conducted digitally. These did not differ in content or length compared to the live interviews. There were 7 focus group interviews, with 3–7 participants in each group. Separate focus groups were carried out with workers, union representatives and managers (Table 1). Focus group participants knew each other as they either worked at the same workplace or worked for the same union. The interviewers therefore paid close attention to the group dynamic and existing informal or formal power relationships. The interviews were semi-structured, and the interview guide (Supporting information) contained open questions about demands at work, social climate at work, experiences of mental health problems and suicide in the workplace and perceived risk factors for mental health problems in the construction industry. The interviews were conducted by two licensed psychologists KA and MS, who had experience of occupational health research. Some of the the focus group interviews were conducted by both psychologists, during which one led the interviews and the other had an observer role, and some focus groups due to logistic reasons were only conducted by one person.

The interviews took place at either the participants’ workplaces, at the university or via a digital meeting. The length of the individual interviews varied between 37 minutes to 60 minutes, with an average of 50 minutes. The length of the focus-group interviews varied between 42 minutes to 77 minutes, with an average time of 66 minutes. Interviews were tape-recorded and professionally transcribed verbatim.

### Data analysis

Thematic analysis [26] was used, as this was deemed suitable to explore perceptions of mental ill health, suicidal behaviour and working conditions among male construction workers. Thematic analysis is theoretically flexible and can be applied using different ontological and epistemological approaches. In this study a realist approach was adopted, meaning that it was considered possible to explore the participants’ experiences of their reality via interviews [26]. It was assumed that the investigation of mental ill health and suicidal behaviour among male construction workers could result in a number of themes rather than being explained by one single phenomenon. Hence, we aimed to present a description of the entire dataset relating to the research topic. This is suitable when there is less knowledge and previous research on the topic, as described by Braun and Clarke [26]. An inductive, data-driven approach was chosen as it would enable the identification of patterns and unexpected themes, which would provide a rich, detailed, and multifaceted account of the underlying data, not preconceived by the researchers. However, a reflexive attitude was adopted and critical discussions attended to the context and considered our pre-existing knowledge.

The analysis was performed by one of the authors (KA) according to the six phases described by Braun and Clark [26]. The first and second steps were to get familiarized with the data by reading each transcript several times, while registering initial codes that captured interesting features of the data. The entire dataset was systematically coded. In a third step, emerging conceptual themes were identified. Themes were identified based on their “keyness”, described as capturing something important in relation to the research aim. Next, the list of main themes was reviewed and refined until a list of defined main themes and sub-themes was established, capturing coherent data to create mutually exclusive themes. In order to strengthen trustworthiness, one of the co-authors (MS) also read all the interviews and checked and then discussed the coding done by KA, until a list of themes was agreed on. Then three of the other co-authors reviewed the analysis and slight changes were performed until a final list of main themes and sub-themes was agreed on. The final themes were named and defined, and specific quotes from the interviews were selected to describe each theme. See Table 2 for an example of the analytical process.

**Table 2:** An example of the process of abstraction.

Unit of analysis	Code	Sub-theme	Main theme
*“It is still a little taboo to admit that I am depressed*, *or that I feel that I have totally lost my footing”*	Feels it is taboo to talk about depression/mental health, does not want to share problems	Stigma related to mental health problems	Difficult to talk about mental health

### Ethics

This study was approved by the Regional Ethical Review Board, Linköping, Sweden (EPN Linköping Approval number—Dnr 2022-01725-01). Informed consent was obtained with a written form signed by the participants before the interview started. In the event that any interviewees became distressed during the interview process, the interviewers were able to provide professional support and referral to further assistance as needed.

## Results

The analysis of the data resulted in identification of five main themes and a number of sub-themes (Table 3).

**Table 3:** Main themes and sub-themes.

Main themes	Sub-themes
1.Difficult to talk about mental health	**1.1 Stigma related to mental health problems** *“There is probably not a lot of talk about mental health in the shed [place for taking breaks]*. *Unless you count stress… and often you wouldn’t say “I am so stressed”*, *you would say it in a different way like “Damn*, *we are under a lot of time pressure*.*” Union representative* **Macho culture** *“The jargon here is tough… it is a male occupation and there´s a lot of swearing” Construction worker* **Suicide–out of the blue** *“When my colleague took his own life… he didn’t show any signs at all*.” *Union representative*
2. Demanding working environment affects mental health	**2.1 Stressful industry** *“The stress is getting worse and worse each year*. *Less time allotted for construction”*. *Construction worker* **2.2 Physical pain leads to psychological pain** *“There is a lot of physical stuff… and what is tragic is that it may start with a bad back that leads to mental health problems*.*” Union representative* **2.3 Hierarchical organisations** *“There is a form of insider culture… and you don’t mess with the workers who are already established”*. *Construction worker*
3. Substance abuse among construction workers	**3.1 Alcohol and suicide** *“When I think back there is a red thread among those who have taken their lives; it is alcoholism and old age*. *I specifically think about a colleague who was retired when he killed himself*, *and according to his son he did it because of chronic work-related pain*.*” Construction worker* **3.2 Overuse of painkillers** *“Stress is a risk factor…If there is dust you can wear a mask*, *but if there is stress you cannot… what do you wear to avoid stress*? *And then there are painkillers and alcohol to ease the whole thing*.*” Construction worker*
4. The importance of management	**4.1 Managers miss mental health issues** *“I have not experienced any direct support*. *Even if I have been open*, *I have not said straight out that I don’t feel well*. *I have sent signals that I don’t feel on top of the world*. *But the response has been”lets deal with that later*, *we will finish these projects first”*. *And then there are new projects*.*” Construction worker* **4.2 Supportive managers make a difference** *“I got support from everyone*, *from the highest manager…It meant a lot to me*, *that I am safe*, *I am not alone” Construction worker*
5. Need for routines and social support in the workplace	**5.1 The need for routines about mental health** *“We don’t have any… clear instructions on how to do it [get help]*. *There is nothing like that*, *so I assume that you would speak to the colleague that you feel closest to*.*” Construction worker* **5.2 Social support from the collegial group** *“I think that if you dare to say that you don’t feel well*, *then there are a lot of hands*, *or hugs*, *that are waiting*.*” Union representative* **5.3 The role of occupational health services** *”Sure we have occupational health care*, *but in a psychosocial sense we don’t have occupational health care*. *It was just nonsense*, *I didn’t get any help from there”*. *Construction worker* **5.4 Wish for more support from the union** *“The trade union should focus more on these questions [psychosocial]*. *If the trade union put pressure on the companies*, *then the companies would have to discuss these questions*.*” Construction worker*

### 1. Difficult to talk about mental health

Many participants described that it was difficult to share personal experiences of mental health problems in the workplace. There appeared to be a stigma in relation to mental health problems, and an attitude that one should not burden others with personal problems. Workplace suicide came as shocking surprise to the colleagues.

### 1.1 Stigma related to mental health problems

Many participants reported that it was difficult to talk about painful emotions in the workplace. Mental health problems were not shared with colleagues.

*“It is still a little taboo to admit that I am depressed, or that I feel that I have totally lost my footing”. Union representative*

*“You can joke and pound someone’s back and say “I am sure it will be ok”*. *But a deeper conversation than that*, *I have never experienced that someone in the workplace has said that they are feeling unwell*, *it just has never happened”*. *Union representative*

When individuals were having mental health problems it was easier to say that that they were suffering from something somatic.

*“I think that you blame something else [instead of mental health problems]… It is easier to say that “Shit my knee is dislocated or I broke my back, or something like that.” Manager*

#### 1.2 Macho culture

The construction industry was often described as being as being dominated by a macho culture. The communication style was tough, with a fine line between jokes and insults. Derogatory terms were used among colleagues, and there was a culture of not complaining about feeling tired or being in pain.

*“It is old fashioned jargon that is still used. When you are at home taking care of a sick child someone says “Don’t you have a wife at home?” Of course, you should be at work even if you are ill.” Construction worker*

*“There is an attitude that implies that you should be in a certain way*, *I don’t want to repeat what is said at the building sites*, *but it is very rough”*. *Construction worker*

#### 1.3 Suicide–out of the blue

Participants who had experienced that a colleague at work had taken his own life, described that it was very unexpected. The colleague had not shown any signs of feeling low or suicidal according to the participants. They also said that it had been a shocking experience and that it had been difficult to understand what had happened.

*“I had a friend, who unfortunately is not alive anymore, who came to me as an apprentice. We worked closely together, and I did not notice anything, and I talked to others afterwards and nobody had understood, could understand that he was as bad off as he was.” Construction worker*

*“Then we had a suicide that hit our group*. *Bang… and then there was a guy who killed himself*. *Nobody knew anything*, *nobody had noticed anything*, *nobody had noticed anything at all*. *How the hell could this happen*!*” Union representative*

### 2. Demanding working environment affects mental health

The working environment in the construction industry was described as risky, physically demanding and stressful which was perceived to have a negative effect on both physical and mental health. Many participants reported that the construction industry was becoming increasingly stressful, with successively shorter deadlines. One effect of the demanding working environment, according to the participants, was long-term pain, which could lead to mental health problems.

#### 2.1 Stressful industry

Most of the participants described that it was a stressful industry. The demands were constantly increasing, and it was described that the pressure affected mental health.

*“They don’t give a shit about the fact that it takes one hour to install a plaster board, it should take 30 minutes. And then you get a time schedule that is so tight that you don’t meet it most of the time. It becomes shit… you have to work faster and in the end you get burned out, go on sick-leave, injured… it is a heavy industry.” Construction worker*

#### 2.2 Physical pain leads to psychological pain

Work related pain was described as a common problem in the construction industry, and several of the participants believed that this long-term pain had a very negative effect on mental health and lead to psychological pain.

*“I think that the most common thing in our industry… it is that you have a physical illness that in the end becomes mental illness”.” Union representative*

#### 2.3 Hierarchical organisations

The organisations in the construction industry were very hierarchal according to participants. Within this system it was important to know one’s place in the hierarchy and respect superiors. This was particularly apparent for trainees who were at the low end of the hierarchy.

*“You notice the hierarchies, it is a typical thing in the construction industry, “I am the oldest, I know best, and I don’t care what you think”. Manager*

There were many descriptions of a bullying culture particularly directed towards apprentices and newcomers to the industry.

*“We all carried two plasterboards; that is heavy enough. But when the apprentice arrived, he had to carry three. But no one said anything, and we laughed at him carrying those boards because they were really heavy. Everyone did that and I was part of it. It had been done to me, so I did it to others. I also thought it was very funny. In hindsight I can reflect back on this and think—what kind of culture is this, what kind of attitude, what kind of group, what does it do to people?” Union representative*

### 3 Substance abuse among construction workers

The construction industry was connected to excessive use of alcohol, which was sometimes used for the purpose of self-medication and this was described as a problem by many participants.

#### 3.1 Alcohol and suicide

Several participants who had suffered the loss of colleagues by suicide, believed that alcohol was part of the problem. Excessive use of alcohol was viewed as a warning sign that someone was not feeling well.

*“I have a colleague… I didn’t work that closely with him, but I got to hear afterwards that it was the alcohol that made him feel bad and take his life. And that is a factor that I have seen before. For the workers who work away from home, then it is more acceptable to drink.” Construction worker*

#### 3.2 Overuse of painkillers

Long-term pain was viewed as a serious problem in the construction industry. The overuse of painkillers, as a way to cope with the pain, was described by several participants.

*“When you sit at home alone you suppress the pain and become addicted to painkillers, you start to self-medicate.” Union representative*

### 4. The importance of management

It was suggested that managers could play an important role both in the exacerbation and mitigation of mental ill health. The managers’ attitudes affected the workplace culture and there were many examples of managers failing to see signs of mental health problems in their employees. However, there were also many examples of how empathic managers had played an important part in supporting employees when they were struggling with mental health problems.

#### 4.1 Managers miss mental health issues

Many participants shared examples of how managers had failed to see and act when employees showed signs of mental health problems. According to the participants managers had failed to provide support in situations where this was needed.

*“When I signal that I don’t feel well, then the management can say “But what the hell, bite the bullet, or leave the industry. You don’t have to be here”. Union representative*

Participants pointed out that it can be difficult to understand when someone is not feeling well.

*“You have good managers and bad managers. Sure, some can see the signals, but at the same time it is difficult when you hardly know yourself.” Construction worker*

#### 4.2 Supportive managers make a difference

Supportive and empathic managers could make a big difference for employees’ mental health according to the participants. A manager who had been supportive when employees had problems that could affect their wellbeing was very appreciated.

*“And then I hit the wall so to speak. So, my manager he brought me home. We came here, we sat and talked and he was very committed to get me back to work. Because I was newly employed it would have been easy for him to ditch me, thinking this will not turn out well. So, he should be praised… he lost a fair number of hours going to my home and staying for two hours to talk. And then he took care of me and said, “Now you and I will work side by side for a week”. So, I feel that he saved me.” Construction worker*

Managers also played an important role in creating a culture in which employees dared to talk about how they were feeling, and a culture where negative behaviours were not accepted.

*“Something that is incredibly important for daring to talk is the company culture. And the workplaces where I have been where this has worked well, the reason for this has been a manager that believed that these [psychosocial] questions are important.” Union representative*

### 5. Need for routines and social support in the workplace

Many participants expressed that it was unclear to them where and how they could seek help if they, or a colleague, were experiencing mental health problems. This lack of knowledge was viewed as a problem and there was a strong wish for information regarding how to get support. Occupational health services were not always viewed as a resource in this context. However, several participants suggested that such services had the potential to provide more support relating to psychosocial factors at work. In addition, the unions had an important role to play in providing support.

#### 5.1 The need for routines about mental health

Participants expressed uncertainty regarding the availability of psychological support in the workplace. There was a wish for more information. Participants wanted clear and specific information regarding what employees could do if they themselves or a colleague needed psychological support.

*“I think that the company should be more explicit about this [where to get help]. But that goes for all companies and workplaces, that if you feel unwell you can do this”. Construction worker*

Several participants suggested that it was important that the information was openly displayed around the workplace.

*”They can write that if you feel unwell, ok you can call this number. And place it by the door or next to the work computer”. Construction worker*

#### 5.2 Social support from colleagues

Participants described how the workmates could provide good social support. However, this support was only available for individuals who belonged to the group. If you did not, you lacked social support at work.

*“I think that if you are a close group, then it is very common for men to show that you are strong, loyal and helpful. But if you are not part of the gang then it is probably more of a burden to be there, that you have to solve your own shit”. Manager*

*“When you finally win respect*, *it is often more loving compared to other types of workplaces*. *Definitely*. *You don’t leave one of your own behind*. *But first you must show that you are a worthy group member*.*” Manager*

Some participants related that poor work performance could be a reason why some individuals did not belong to the group. And the poor performance could be caused by poor health.

*“It is important to deliver, and the one who fails to deliver becomes difficult to handle. And looking back I was part of creating that culture, I liked that culture. Why? Well, I was never vulnerable. But I can see that some of the people I worked with did not feel that well. And then I can think about what was wrong with us…? We lacked knowledge, and we lacked a language to deal with colleagues who did not feel well.” Union representative*

#### 5.3 The role of occupational health services

It was clear that many believed that occupational health services could play a greater role in supporting employees with psychological problems.

*“Perhaps it is a bit easier to open up at the Occupational Health Services. Because you go there every other year and it is a good opportunity. I don’t think it [the industry] is so damn macho. Well, of course you don’t want to talk, but if you get the chance to talk to someone, I think it will become easier for many… And you don’t want to book the appointment yourself.” Construction worker*

However, there were also disappointments relating to occupational health services. One reason was that the support had to be approved by the manager, and if the manager did not approve this, it could feel like ones’ value as a person was being judged in a negative manner.

*“You had to get the appointment approved by the management, and then it becomes a question about power… And it is not good that I should have to beg to get that appointment and perhaps get turned down, then you feel really bad. Am I not worth more than that? So that was the reason to why I did not go down that road.” Construction worker*

#### 5.4 Wish for more support from the trade union

Several participants highlighted that the trade unions could help to increase the focus on psychosocial issues at work.

It was suggested that the union could play an important role as a third party, when the employees did not want to speak directly to the employer. One suggestion was for an emergency phone line, where employees could call when needed.

*“I suggest that the trade union should have some form of emergency phone line… It would mean a lot to have a third party…Because if you have to sit there and explain to your manager why you have not said anything previously. Of course, you grit your teeth (and don’t say anything)”. Construction worker*

For some employees the union was a first point of call when they needed help. According to the participants who worked at the unions, it was difficult for the union representatives to talk about mental health as they had no training.

*“As a union representative I was an expert at putting on band aids and help them if they had banged their head and were bleeding. The physical issues were not a problem. But when the macho culture is strong and you should not show any weakness, and a demolition worker came and said that he had a really difficult time in his relationship and that he was worried about his kids… Total silence, where do we have the tools to deal with this conversation?”Union representative*

## Discussion

The current study explored perceptions of mental ill health, suicidal behaviour and working conditions among male construction workers, in order to increase the understanding of these phenomenon and to identify relevant avenues for workplace interventions. Five main themes were found in the analysis: Difficult to talk about mental health, Demanding working environment affects mental health, Substance abuse in the workplace, Importance of management, and Need for routines and social support in the workplace. Below, each theme will be discussed in turn.

The first main theme, *Difficult to talk about mental health*, highlighted stigma related to mental health problems in the construction industry. It was perceived to be very difficult to talk about mental health problems and that it was easier to talk about somatic problems. Previous research has found that stigma, relating to mental health, acts as a barrier to help-seeking among construction workers [27]. Similarly, stigma relating to mental health issues and low levels of help-seeking have been reported to contribute to the elevated risk of suicide observed in construction workers [20]. The participants in the current study also described a macho culture that encouraged a narrow gender role for males that promotes self-reliance and does not encourage help seeking or sharing of vulnerabilities. Construction site work has been linked to traditional masculine behaviours such as stoicism, emotional restraint, risk-taking, competitiveness and toughness [28]. It has also been suggested that stigma toward mental health problems may be particularly apparent in male-dominated industries, such as construction, where traditional ideas about masculinity tend to be maintained [27]. A literature review of suicide in the construction industry suggested that the macho culture was one reason for the elevated levels of suicide in this industry [22]. A previous study [21] suggested that many workers in the construction industry do not share that they are distressed and consequently do not identify or seek help to deal with their distress. Wittenhagen and Meurk’s [21] study identified that the construction industry was in need of a shift in workplace culture in order to promote help-seeing behaviour to reduce distress. A supportive non-judgmental environment that respects individual privacy was viewed as very important. The results in the current study highlighted that suicide was viewed as something that occurred out of the blue, and that it was difficult to see any warning signs. Follmer & Jones [29] have suggested that, despite organisations’ best efforts, some employees may not disclose suicidal ideation at work because of fear of stigmatization or discrimination. In the current study the participants were feeling shocked when a suicide had occurred and described being very emotionally affected. Howard et al. [5] found that suicide deaths have negative consequences beyond the individual, affecting co-workers and organizations and suggests that employers have good reasons to attend to how the workplace can provide support after workmate suicide. A review of interventions for suicide survivors [30] found that each suicide impacts many people, and co-workers often experience negative emotional and behavioural consequences, such as social withdrawal and reduced productivity, as a result of the loss. Indeed, some suicide programs include postvention, that entails interventions following a suicide in a work group. The aims of postvention are to alleviate the distress and promote recovery of the affected group of co-workers and to reduce risk of further suicides in the workgroup [9].

The second main theme was the *Demanding working environment affects mental health*. According to the participants the working environment was very stressful and physically demanding and had a negative effect on workers’ wellbeing. This is in accordance with previous research that has found that stressful and physically demanding working conditions could be contributing factors to suicide [5, 7, 9, 10, 12, 31]. In the current study the participants described that long-term physical pain often caused by occupational injury or illness, caused by high physical demands, could lead to psychological pain. Previous research has found a comorbidity between physical pain and psychological disorders such as depression and anxiety [32]. Construction work is physically demanding and can be challenging and dangerous which may lead to both musculoskeletal disorders and occupational injuries [27]. These physical risks may lead to both physical and mental health problems [4]. Occupational injuries are associated with elevated rates of suicide, which can be mediated by pain, loss of work role, reduction of income, adverse experiences with disability support processes, and more [9]. In the literature review of suicide in the construction industry risk factors included physical injury and chronic pain [22]. In the current study the participants also reported a hierarchical culture in the construction industry where particularly apprentices were exposed to a lack of support and at times bullying. Similar findings have been reported in previous research. For example, one Australian study found that apprentices are faced a number of challenges while transitioning into the construction industry; they needed to learn their unique position in the social and organisational hierarchy [33].

The third main theme was *Substance abuse among construction workers*. Excessive use of alcohol was perceived as a strong risk factor for suicide. Alcohol and substance abuse has been identified as a risk factor for suicide in the construction industry [22]. It has been estimated that alcohol is involved in approximately 30% of all suicide attempts [34] and alcohol use is a substantive risk factor for death by suicide [35]. Previous research has found that substance abuse problems are more common among construction workers compared to members of other occupational groups [27]. In the current study overuse of painkillers was described as a way to cope with long-term pain, and as a risk factor for suicide. Previous research has found that using painkillers to alleviate work-related pain is a common problem in the construction industry [36, 37]. It has also been suggested that it is not uncommon that workers in the construction industry self-medicate with alcohol or medication instead of seeking help when feeling distressed [21].

A further main theme was the *Influence of management*, which highlighted that managers may play an important role in both catalysing and alleviating mental health problems. Some managers with low mental health literacy missed typical signs of mental health issues. A recent study on distress in the construction industry found that some individuals may hide their distress, thus requiring that someone else notices the signs [21]. A systematic literature review of work and suicide [4, 5] found some support for the relation between leadership constructs and suicidal ideation, but pointed out that this research is still in its infancy. Previous research has found that interventions aiming to improve leaders’ mental health awareness have been effective in increasing intentions to promote mental health at work [38, 39], and this is an explicit action recommendation in the WHO Guidelines on work and mental health [40]. The MATES programme from Australia, a multimodal workplace-based suicide intervention program for the construction industry, aims to train individuals at all levels in an organisation including managers [41]. A systematic review of the MATES program found that it was effective in improving mental health and suicide prevention literacy, and that peer support workers were the most trusted people to go to with concerns [41]. Hence, mental health support training might most beneficially be offered to workers at all occupational levels, ranging from labourers to union reps and managers.

The final main theme was *Need for routines and support in the workplace*. The results from this study add to existing knowledge by highlighting that many individuals did not know what to do to if they themselves, or a workmate was suffering from mental health problems. A need was expressed for clear routines and guidelines for how individuals could get help and support regarding mental health. Indeed, many have suggested that the workplace is an important arena to tackle mental health problems, regardless of whether work caused the problems or not [5, 9, 31, 42, 43]. Howard et al. [5] suggested that organisations should provide support that can be accessed independently and anonymously, and at a minimum provide information on suicide hotlines and contact information for local counsellors and therapists in order to raise employees’ awareness of available support services. The MATES programme is an example of workplace support that connects workers in the construction industry to the most suitable available help [41]. The study by Wittenhagen and Meurk [21] identified many different options for distressed construction workers to seek help, including work colleagues or supervisor, MATES in Construction, helplines, sports clubs, health professionals, Employee Assistance Programs. In the current study it was suggested that colleagues could provide good support, but that this was only the case for the workers who belonged to the collegial group. In the systematic literature review of work and suicide by Howard et al. [5] workplace social support was found to be associated with a lower risk of suicide. It was also suggested that being excluded or ignored at work, may have a particularly strong effect on suicidal ideation, as this behaviour may lead to extreme feelings of thwarted belonginess [5, 11]. A study of the French working population found associations between suicide and low social support [12]. In the current study the participants expressed that the occupational health services could play an important role in providing support in the workplace, but that this was not always the case. For example, one issue was the need for approval by the manager before the service could be accessed. In the study by Wittenhagen and Meurk, [21] it was found that some individuals may not seek help because they did not trust the services provided through the workplace. Some workplace suicide prevention programs, internationally, provide independent sources of support that are confidential and do not require management approval [40].

### Strengths and limitations

Focus groups provide a useful and flexible method that takes advantage of the communication between the participants. However, the option of individual interviews was also provided considering the sensitive research topic. Thus, it can be a limitation that both focus group and individual interview data were included. In the analysis of the data attention was paid to apparent differences in the content, between group and individual interviews, and it was judged that all interviews could be analysed as one data set. Moreover, it may be possible that the participants found it difficult to freely express their experiences and opinions in the interviews, particularly in the focus group interviews. The interviewers informed the participants that there was no need for consensus in the interviews, that all experiences and opinions were welcome, and that full confidentiality outside of the group was ensured. Another limitation is that six of the individual interviews were conducted digitally, and it is possible that the digital format influenced interview content. However, the interviewers judged that the digital interviews did not differ in length or content compared to the live interviews. A strength of the study was that the participants were workers, managers, or union representatives from different organisations, which contributed to a variety of descriptions of the perceptions of mental ill health and suicidal behaviour among male construction workers. A further strength of the study was researcher triangulation. Two researchers were involved in data collection and analysis, which increased the trustworthiness of the findings [44]. Moreover, the researchers held continuous reflexive discussions throughout the study [45]. Regarding transferability of the findings, it is possible that the themes can be relevant to other individuals and workplaces in the construction industry in similar settings. While it is not the intention to make generalisations from qualitative studies, it is important to relate the findings to previous research and thereby add to the accumulation of results on the topic under study [46].

### Practical implications

The findings of the current study highlight several aspects that could be important for future suicide prevention and promotion of psychological wellbeing at work.

- Talk openly about mental health—facilitate discussions about mental health in the workplace in order to decrease the stigma and encourage help offering and help seeking. This could be done by providing information about mental health and systematically highlighting the topic in different situations at the workplace. However, it is also important to facilitate confidential support when this is needed.
- Change the macho culture—organisational culture is not quickly or easily changed, nevertheless an implication of the findings is that the macho culture appears to be a contributing factor to why it can be difficult to admit to vulnerability and seek help. Changing the culture relates back to the previous suggestion of facilitating open discussions about mental health, which would ideally involve both workers and managers.
- Training—provide training on mental health and suicide for managers, workers, union-representatives and other stakeholders. The training could include recognition of risk factors in the working environment, signs of mental ill-health, and provide support for how to talk to individuals with depression and suicidal issues.
- Clear routines regarding support–provide routines and easily assessable guidelines about the available support for mental health problems. The guidelines should include information about occupational health services, other health services and the services that can be provided by the unions. Provide information about support that is confidential and does not require approval from the employer. Workplaces should also offer help via occupational health services including counselling, screening and treatment for alcohol and drug problems, and screening and treatment for chronic pain.

These recommendations are not novel but can be seen as a suggestion for best practice. The suggested actions have been addressed in existing suicide prevention programs such as MATES in Australia [32]. Recent research has suggested that interventions can be helpful in decreasing suicide rates among male construction workers [13]. And these recommendations, which fit well with previous research, could be useful for future interventions.

## Conclusions

The results from this Swedish study fit well with previous international findings regarding the challenges of a macho culture and persistent stigma of mental health in the construction industry. The themes also described a demanding working environment, a link between physical and psychological pain and alcohol as a serious risk factor for suicide. The study adds to existing knowledge by highlighting that suicides that had occurred among workmates were perceived to be very unexpected, and that many individuals did now know what to do to if they themselves, or a workmate was dealing with mental health problems. Possible implications for workplace interventions include facilitation of discussions of mental health in the workplace, training at work to recognise risk factors and to talk about mental health problems and clear routines in the workplace regarding support for mental health problems.

## Supporting information

S1 File (DOCX)
