# Supplementary material for: Perceptions of mental health, suicide and working conditions in the construction industry—A qualitative study
Source: PLoS One. 2024 Jul 24;19(7):e0307433. doi: 10.1371/journal.pone.0307433 (PMC11268583; doi:10.1371/journal.pone.0307433)
Supplement: S1 File — (DOCX) [file pone.0307433.s001.docx]

**Interview guide**

The interviewer presents themselves, presents the agenda for the interview and goes through the confidentiality section of the informed consent form.

**Brief**  presentation of participants and their main work tasks

**Demands and work**

- The physical demands can be high in the construction industry, but are there work tasks that can be psychological demanding/how/why/examples
- Can those factors affect well-being at work/outside work/examples
- Are/how can those factors be dealt with/ is this done/examples

**Social climate at work**

Do you talk about mental health at work/how/when/examples

Are there situations that encourages more personal conversions/when/examples

Do you/others at work pay attention to if work mates are suffering from mental health problems/examples

Have you experienced that work mates that are dealing with mental health problems have been criticized or bullied because of this/examples

**Mental health and suicide at work**

- Does your employer work with improving mental health at your workplace/ how/examples/ is the work systematic?
- What help is available at work for mental health problems/examples/are these services used/well known?
- What more could be done to prevent and handle mental health problems at work?
- Have you any experience of a work mate being suicidal/ how would you handle this/ how would/was this handled at the workplace/?

**Risk factors in the construction industry**

- What are the biggest risk factors for mental health problems in the construction industry/why?
- Are there risk factors that are more common in the construction industry compared to other industries?

Is there anything that you want to add that we have failed to ask about?
